# Supplementary material for: Learning curve and surgical outcome of robotic assisted colorectal surgery with ERAS program
Source: Sci Rep. 2022 Nov 29;12:20566. doi: 10.1038/s41598-022-24665-w (PMC9709162; doi:10.1038/s41598-022-24665-w)
Supplement: Supplementary file 1 — Supplementary Information 1. [file 41598_2022_24665_MOESM1_ESM.zip › LSC Raw data-Ñ[▒K/054/OP N_ñw╝╨░O▒Kñσ.pdf]

W83 / W83

臺中榮民總醫院

手術室護理記錄

[redacted]

基本資料

- 室別： 二醫手術室-31室，手術科別： CRS，手術類別： 常規，日期： 2019/11/05
- 進入時間： 1325 ； 麻醉開始時間： 1325 ； Time Out時間： 1430 ； 劃刀時間： 1431 ； 手術結束時間： 1840 送出時間： 1845 ；
- 麻醉方式： GE

  診斷：sigmoid colon tumor.

  手術名稱：1.Laparoscopic Anterior resection and anastomosis - malignant
  2.Laparoscopic Cholecystectomy

  手術局部用藥：NIL

  術前預防性抗生素：
    - 抗生素名稱一： Veterin 1GM IVD(由麻醉科護理人員給予)

  ，追加預防性抗生素：
- 手術醫師： 林俊余，吳峰旭，許家榮，黃俋霖，林陳彥
- 麻醉醫師： VS 施涵薰
-
    - 刷手護士A ： 羅渝軒，時間： 1325-1730，接替刷手一： 楊智雯，時間： 1730
    - 流動護士A ： 吳雅華，時間： 1325-1530，接替流動一： 洪紹瓊，時間： 1530-1800，接替流動二： 羅渝軒，時間： 1800

皮膚狀況

A-V Shunt： 無

術前： 完整

記錄者： 上次簽章: 吳雅華

術後： 完整

記錄者： <u>羅渝軒</u>

手術準備

手術臥式 單一手術臥式

-
    - 手術臥式： 截石臥式
    - 使用附件： 矽膠枕頭，腳架
    - 皮膚準備： 10%Aq Betadine，Hibiscrub，其它： 2% Chlorhexidine
    - 電燒： 使用電燒: 電極片位置： 其它： 迴路電燒墊，記錄者： 上次簽章: 吳雅華

止血帶T： 無

植入物

無

標本送檢

- 細菌培養
- 細胞學 無
- 冰凍切片 無
- 病理標本

  組織名稱一：GB

  ,

  組織名稱二：S-colon

  ,

  組織名稱三：distal cutend

  ，更多組織名稱
    - 組織名稱四：IMA Root
- 截肢 無

標本交付 標本： 肆 袋，標本： 零 盒，標本： 零 瓶，細胞學： 零 管，Culture Tube： 零 管，被交付醫師： R 林陳彥，

- 記錄者： 羅渝軒
- 時間： 1840

傷口術後數料

傷口數料：　　　　　　　　　　傷口數料： 無

- 請於圖上標示部位：

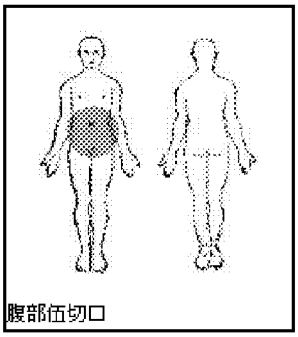

- 傷口數料： mepore

導管

導管	天數	導管資訊	插入日期	插入時間	更換日期	拔除日期	拔除時間	拔除原因	預計拔除/更換	預計拔除/更換日期
Foley(1)	1	號碼:16 Fr.; 部位:膀胱;	2019/11/05	1400						
Sump NG Tube(1)	1	號碼:16 Fr.; 深度:60CM; 部位:胃;	2019/11/05	1410						
Penrose Drain(1)	1	號碼:12 mm.; 部位:骨盆腔;	2019/11/05	1735						
A-line (1)	1	號碼:20 G; 部位:前臂; 象限:LU;	2019/11/05	1405						

用物計數

- 手術中出血量： 微量 c.c.
- 手術中尿量： 900 c.c.
-
  - 其它： 無

用物計數： 多切口同時縫合

- 1.器械： 正確，2.縫針： 正確，3.刀片： 正確，4.紗布： 正確，5.腹部墊： 未使用，6.紗球： 未使用，7.PEANUTS ： 未使用，8.紗條： 未使用，9.絲帶： 未使用，10.針頭： 未使用，11.Vessel loop： 未使用，12.Boots： 未使用，13.Jelco： 未使用，14.棉條： 未使用，
  - 第一次：
    - 刷手護士 ： 羅渝軒
    - 流動護士： 上次簽章: 洪紹瓊
    - 時間： 1730

  ,
  - 第二次：
    - 刷手護士 ： 楊智雯
    - 流動護士： 上次簽章: 洪紹瓊
    - 時間： 1740

  ,
  - 第三次：
    - 刷手護士 ： 楊智雯
    - 流動護士： 羅渝軒
    - 時間： 1800

備註

備註：NIL

- 記錄者： 上次簽章: 洪紹瓊
- 時間： 1617

填寫護理人員_羅渝軒________________

正式摘要

		W83 / W83			

doctorRank:B
